# Peroxydisulphate activated FTO-WO_3_ nanorods based photoelectrocatalytic degradation of tetracycline: Intermediate products, degradation pathway and ecotoxicity studies

**DOI:** 10.1016/j.heliyon.2023.e20882

**Published:** 2023-10-12

**Authors:** Babatunde A. Koiki, Omotayo A. Arotiba

**Affiliations:** a Department of Chemical Sciences, University of Johannesburg, South Africa; b Centre for Nanomaterials Science Research, University of Johannesburg, South Africa

**Keywords:** Tungsten trioxide, Sulphate radicals, Photoelectrocatalytic degradation, Tetracycline, Toxicity, Water treatment

## Abstract

This work reports sulphate radical assisted photoelectrocatalytic (SR-PEC) degradation of tetracycline using a visible light active fluorine-doped tin oxide – tungsten trioxide nanorods (FTO-WO_3_ NRs) photoanode. The WO_3_ NRs were synthesised via the hydrothermal method and then conducted on the FTO glass to form a photoanode. When the photoanode was applied without sulphate radicals for PEC degradation, 10 % of the tetracycline was degraded. Conversely, when 3 mM persulphate was added, the extent of tetracycline degraded was 88 % using the UV–vis spectrophotometer and 99 % using the ultra-performance liquid chromatography mass spectrometer (UPLC-MS) within 90 min at 1.5 V. The mechanism of tetracycline degradation was proposed based on the intermediate products identified using UPLC-MS and the extent of toxicity was evaluated using quantitative structure activity relationship (QSAR) analysis. Trapping experiment revealed that the photogenerated holes, sulphate radicals, and hydroxyl radicals were the oxidants that significantly took part in the degradation of tetracycline. Overall, the electrode was stable and reusable, therefore suggesting the suitability of FTO-WO_3_ NRs photoanode in the presence of sulphate radicals towards the decontamination of water laden with pharmaceutical pollutants.

## 1 Introduction

In the last decade, the photoelectrocatalytic (PEC) degradation process has received wide acceptance having distinguished itself as a promising and efficient technology towards the treatment of water contaminated with emerging organic contaminants. This is because of the in situ production of hydroxyl radicals widely known to be non-specific in action, with the ability to completely break down myriads of pollutants in water [1]. Harnessing the oxidising power of hydroxyl radicals in the abatement of organic pollutants has proven to be an efficient approach to wastewater remediation. In a fast-growing world of industrialisation accompanied by an increasing rate of emerging toxic, recalcitrant and non-biodegradable pollutants like pharmaceuticals and personal care products, endocrine-disrupting chemicals, polychlorinated biphenyls, etc. in our water sources, hydroxyl radical based PEC degradation may no longer be able to efficiently mitigate the enormous challenges at hand [2]. This is because of its low redox potential (E_o_ = 1.8–2.7 V vs. NHE), as it may not effectively degrade the class of pollutants stated above [3]. Also, due to its short lifetime of about 40 μs, it will be rapidly used up in the system [4]. In as much as necessity drives every research and the goal is always to improve on the performance of an existing wastewater treatment technique. Hence, the distinguishing feature of this work comes with the addition of sulphate radicals into the system to enhance the PEC degradation of antibiotics in water.

Worthy of note is the fact that sulphate radicals possess striking merits such as longer life-time of around 300 μs? This is almost 8 times longer than hydroxyl radicals, thus suggesting that it will survive longer than the hydroxyl radicals in the system and significantly contribute towards degrading more pollutants in water. Also, sulphate radicals possess a redox potential, E_o_ = 2.5–3.1 V vs NHE which is higher than hydroxyl radicals [5]. This implies that the sulphate radical possesses stronger and higher oxidising power that is sufficient enough to break down stubborn organic pollutants. In addition, sulphate radicals possess higher selectivity and reactivity towards the degradation of organics compared to hydroxyl radicals [6]. This shows that as we move towards versatility and sustainability, effluent containing myriads of pollutants can be efficiently treated by sulphate radicals enhanced PEC (SR-PEC) degradation technology as compared to hydroxyl radical PEC degradation technique. These advantages confer on SR-PEC the potential to perform greater than the well-known hydroxyl radical-based PEC degradation system. However, it is quite surprising that only a few works on SR-PEC have been reported.

Visible light active semiconductor photoanodes such as Bi_2_WO_6_ [7], Cu_2_O [8], BiVO_4_ [9], γ-BiMoO_6_ [10], etc. have been reported in literatures to activate persulphate or peroxymonosulphate salts in a bid to generate sulphate radicals for enhanced photoelectrocatalytic degradation of organics in water. Considering the promising advantages associated with sulphate radicals, more visible light active semiconductors must be sought to activate persulphate salt, thereby generating sulphate radicals for improved breakdown of emerging pharmaceutical contaminants in water.

Tungsten trioxide (WO_3_), a n-type semiconductor is known to absorb in the visible light region and possesses a band gap energy within the range of 2.6–2.8 eV. It is cheap, non-toxic, and not easily susceptible to photocorrosion [[11], [12], [13]]. It has been broadly applied in areas such as gas sensing [14], water splitting [15], energy storage [16], perovskite solar cells [17], photocatalytic and photoelectrocatalytic degradation of pollutants [[18], [19], [20], [21]], etc. Therefore, we hypothesise WO_3_ to be a suitable semiconductor material for PEC degradation of pharmaceutical contaminants in water in the presence of sulphate radicals.

Antibiotics are ranked as one of the emerging pharmaceutical pollutants globally due to the unprecedented demand for them and the progressive development in the pharmaceutical industry at large [[22], [23], [24]]. Tetracycline, an antibiotic, is one of the highly potent recommended antibiotics leading to its broad application in curative treatment in humans, livestock and the poultry industry as a whole [25,26]. It has been reported as the second most consumed antibiotic in the world because it is cheap and easy to prepare, among other reasons [27,28]. Due to incomplete metabolism in the body of human beings and animals, about 80–90 % still find their way into the ecosystem [29]. However, because of their stability chemically, they are known to be non-biodegradable, hence making it difficult to remove them from the environment. As a result, the need for a highly efficient treatment technology must be sought to completely remove tetracycline from the ecosystem.

In this work, we present a novel pathway for the abatement of tetracycline by using persulphate enhanced photoelectrocatalytic degradation at an FTO-WO_3_ NRs photoanode. Operational parameters including persulphate concentrations and external bias potential were investigated and discussed. Furthermore, we probed the degradation process by determining the intermediate products using ultra-performance liquid chromatography and proposed the possible degradation pathway for the abatement of tetracycline. We used computational techniques to estimate the extent of toxicity of the parent compound and the intermediate products using the quantitative structure activity relationship (QSAR) model.

## 2 Experimental

### 2.1 Materials

Sodium tungstate dihydrate, sodium peroxidisulphate, sodium sulphate, sodium chloride, ethanol, hydrochloric acid, fluorine-doped tin oxide coated slide (specification: 50 mm × 50 mm x 2.5 mm, surface resistivity ∼7Ω/sq) were procured from Sigma Aldrich, South Africa.

### 2.2 Synthesis

#### 2.2.1 Synthesis of WO_3_ NRs

**Fig. 1:**
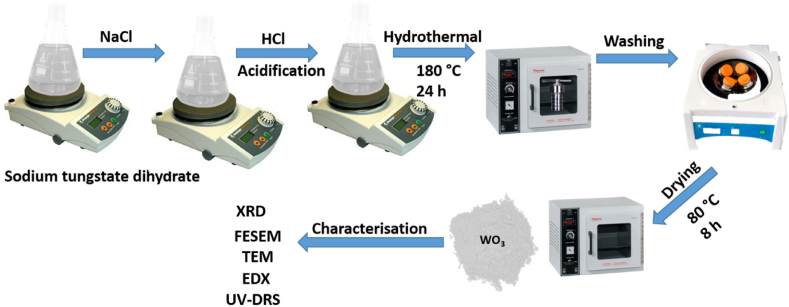
Scheme showing the synthesis of WO_3_ nanorods.

Sodium tungstate dihydrate was dissolved in 80 mL deionised water with further addition of sodium chloride. The resultant solution was acidified to pH 2.06 using HCl and transferred into a Teflon-lined autoclave. This was further placed into the oven for 24 h at a temperature of 180 °C. The product formed was washed and left to dry in the oven at 80 °C for 8 h [30,31]. The synthesis route employed is represented in Fig. 1.

#### 2.2.2 Synthesis of FTO-WO_3_ NRs photoanodes

The as-synthesised WO_3_ was mixed with 90 μL of N-methylpyrrolidone and 5 mg of polyvinylidene fluoride as a binding agent. The mixture obtained was drop-coated on the FTO glass substrate (2 cm × 2 cm). Furthermore, it was oven dried at 60 °C.

### 2.3 Characterisation

The nature of WO_3_ crystallinity was investigated on PANanalytical X'Pert PRO X-ray diffractometer (XRD) Malvern, United Kingdom. The diffraction patterns were obtained from a 2θ degree angle = 10–90° and a step size of 0.0170° using Cu radiation with a wavelength of 0.154 nm. The generator was kept at 40 kV and a current of 40 mA. The internal structures of WO_3_ were examined with Field emission scanning electron microscopy (FESEM, JEOL JSM-7500F, Japan) and transmission electron microscopy (JEOL 2100 HRTEM 200 V, Japan). The optical properties were studied using UV–vis diffuse reflectance spectroscopy (UV–vis DRS) coupled with UV–vis Spectrophotometer Cary 60 UV–vis spectrophotometer (Malaysia).

### 2.4 Photoelectrocatalytic measurements

Photoelectrocatalytic measurements were carried out on an Autolab PGSTAT204 (Netherlands) potentiostat/galvanostat which is a three-electrode system. The visible light source was the Oriel LCA-100 Solar Simulator (USA) equipped with a 100 W xenon lamp and AM1.5G filter that is capable of producing 100 mWcm^−2^. WO_3_ NRs photoanode, platinum sheet and Ag/AgCl electrode (in saturated 3 M KCl) were used as the working electrode, counter electrode, and reference electrode respectively. Photoelectrocatalytic degradation studies were carried out in a 50 mL quartz reactor containing 5 ppm tetracycline in 3 mM Na_2_S_2_O_8_ solution with a potential of 1.5 V. The extent of tetracycline degradation was monitored using UV–vis spectrophotometer, total organic carbon analyser (Teledyne Tekmar Lotix, USA), and ultra-performance liquid chromatography mass spectrometer (UPLC-MS). The ecotoxicity of tetracycline and its intermediate products were studied by Quantitative Structure Activity Relationship (QSAR) model by using Toxicity Estimation Software Tool (T.E.S.T) Version 5.1.

### 2.5 Ultra-performance liquid chromatography-tandem mass spectrometer (UPLC-MS) analysis

To obtain accurate mass data, we used an ultra-performance liquid chromatography–tandem SYNAPT G1 mass spectrometer (UPLC-MS, Waters, USA). Waters HSS T3 C18 analytical column (150 mm × 2.1 mm x 1.8 mm) was used to carry out the chromatographic separation while the temperature of the column was kept at 60 °C. Binary solvent mixtures consisting of ultra-pure water with 10 mM formic acid (Eluent A) and acetonitrile (Eluent B) were used in the separation process. The method for elution involves 98 % eluent A at a flow rate of 0.4 mL min^−1^ which was maintained for 1 min, then a linear gradient to 2 % eluent A at 6 min. These conditions were kept constant for 1.5 min before they were adjusted to the initial conditions. The analysis time was 10 min and the injection volumes varied between 1 and 10 μL. The SYNAPT G1 mass spectrometer was used under both electrospray ionisation positive and negative modes with a capillary voltage of about 2.5 kV to aid the detection of phenolic and other electrospray ionisation compatible compounds. The scan time, Dalton mass range, source temperature and desolvation temperature were 0.1 s, 50–1200, 120 °C, and 450 °C respectively. The sampling cone and extraction cone remained at 30 V and 4.0 V respectively. The collision gas used was argon while the desolvation and nebulisation gas was nitrogen. The target compounds were analyzed based on corresponding chromatography retention time. The instrument control, collection and analysis of data were carried out on MassLynx V4.1 (Waters, USA) [32].

## 3 Results and discussion

### 3.1 XRD morphological studies

**Fig. 2:**
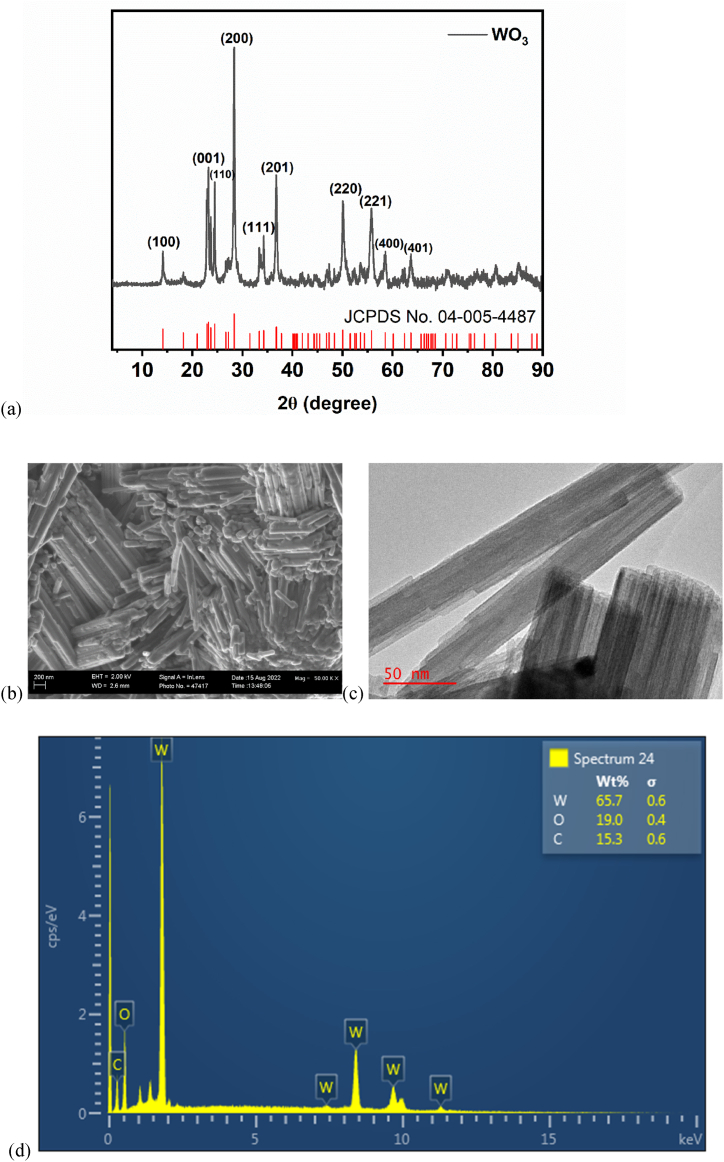
(a) XRD pattern of WO_3_ NRs, (b) FESEM image of WO_3_ NRs, (c) TEM image of WO_3_ NRs, and (d) EDX spectrum of WO_3_ NRs.

The diffraction patterns of the WO_3_ NRs photoanode were examined under an X-ray diffractometer as represented in Fig. 2a. The peaks at 2θ = 14.10°, 22.92°, 24.44°, 28.34°, 33.35°, 36.76°, 50.02°, 55.76°, 58.50°, 63.65° can be indexed as (110), (001), (110), (200), (111), (201), (220), (221), (400) and (401) respectively are characteristic of pure monoclinic WO_3_ (JCPDS Card no. 04-005-4487) [31]. In addition, the diffraction peaks at 2θ = 14.10°, 22.92°, 28.84° and 36.76° corresponding to (110), (001), (200), and (201) planes respectively are suggestive of WO_3_ nanorods [33]. In addition, the crystallite size of the synthesised material was estimated and found to be 46.47 nm.

To investigate the morphology of the as-synthesised WO_3_, morphological studies were conducted on the material using both FESEM and TEM machines, and images were captured as presented in the figures below. The FESEM image represented by Fig. 2b showed a clustered rod-like image which is characteristic of WO_3_ as reported in literatures [31,33]. Further insights into the morphology were gained from the TEM image represented by Fig. 2c. The TEM confirmed the synthesised WO_3_ as nanorods. Hence, we can safely say that our as-synthesised material is WO_3_ nanorods. To corroborate this, the EDX spectra (Fig. 2d) confirmed all the expected elements such as tungsten and oxygen to be present.

### 3.2 Optical properties

To gain insight into the optical behaviour of the FTO-WO_3_ NRs photoanode, the absorption spectra were obtained from UV–Vis DRS. As shown in Supp Fig. S1a, the estimated absorption band edge of WO_3_ was ∼440 nm. This suggests that the synthesised WO_3_ NRs fall within the visible light region and ultimately implies its suitability and efficiency under sunlight. Similarly, the estimated band gap energy from the Tauc plot as shown in Supp Fig. S1b was found to be 2.99 eV. This is in agreement with what we have in literatures [31,34].

**Fig. 3:**
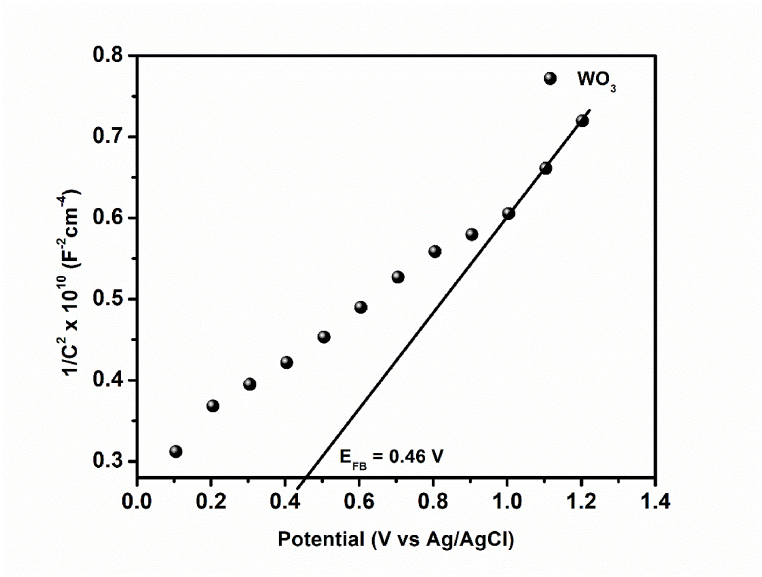
Mott Schottky plot for FTO-WO_3_ NRs photoanode in a 5 mM solution containing [Fe(CN)_6_]^3-/4-^ prepared in 0.1 M KCl solution.

To estimate the flat band potential of the synthesised photoanode, the Mott-Schottky plot obtained from the impedance data is represented in Fig. 3. The positive slope obtained from the plot is indicative of the n-type nature of WO_3_ and the estimated flat band potential (E_FB_) from the intercept fixed at 1/C^2^ equals zero was 0.46 V vs Ag/AgCl. The valence band (VB) and the conduction band (CB) edge potentials of WO_3_ NRs can be estimated using eqns. (1), (2)):

(1) $$E_{FB}=E_{CB}+\Delta E$$

(2) $$E_{VB}=E_{CB}+E_{g}$$

The potential difference between E_CB_ and E_FB_ (ΔE) is −0.1 to 0 eV for an n-type semiconductor [35]. The E_CB_ of WO_3_ NRs was calculated to be 0.56 V vs Ag/AgCl using eqn. (1). Furthermore, given the band gap energy (E_g_) of WO_3_ to be 2.99 eV the E_VB_ was estimated to be 3.55 V vs Ag/AgCl. Interestingly, the E_CB_ and E_VB_ values were almost the same as the values obtained theoretically using the Mulliken electronegativity theory expressed in eqns. (3), (4)):

(3) $$E_{CB}=X-E_{C}\;–\;0.5E_{g}$$

(4) $$E_{VB}=E_{g}+E_{CB}$$

where X represents the electronegativity of WO_3_ normally expressed as the geometric mean of the absolute electronegativities of the constituent atoms with the value 6.59 eV [36], and E_c_ represents the energy of the free electrons on the hydrogen scale with the value 4.50 eV. Using equation (3), the E_CB_ obtained is 0.595 eV and the E_VB_ obtained is 3.585 eV.

### 3.3 Photoelectrocatalytic degradation of tetracycline

**Fig. 4:**
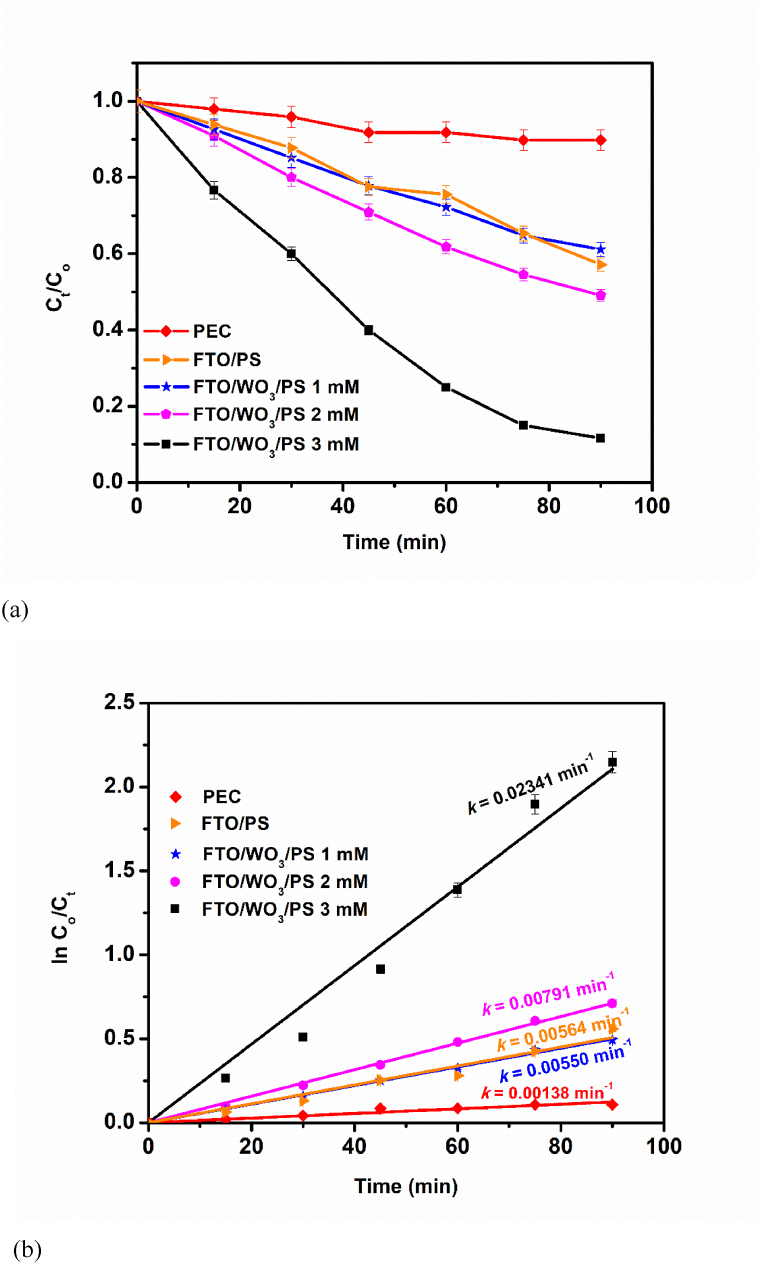
(a) Photoelectrocatalytic degradation plots of tetracycline, (b) Kinetics plots for degradation of tetracycline. Experimental conditions: [Tetracycline] = 5 ppm.

The FTO-WO_3_ NRs photoanode was applied towards photoelectrocatalytic degradation of tetracycline with and without persulphate (PS) as represented in Fig. 4a. In the absence of PS, the PEC removal of tetracycline in the presence of Na_2_SO_4_, the commonly used supporting electrolyte, the extent of degradation was found to be 10 % after 90 min. On introducing 1 mM PS, the extent of tetracycline degradation rose to about 39 % within 90 min which is almost 4 times the result obtained in the PEC system without PS. This notable increase can be ascribed to the presence of the sulphate radicals produced by the addition of the persulphate. It further confirms the improved performance of the sulphate radicals in an SR-PEC technique compared to the hydroxyl radicals in the well-known PEC system. In addition, an increased extent of degradation was observed as the PS concentration increased from 2 mM (51 %) to 3 mM (88 %) after 90 min. This enhanced PEC performance is as a result of the existence of more sulphate radicals that are been produced by the activation of PS by FTO-WO_3_ NRs photoanode. The increase in the rate of degradation because of the addition of PMS has been reported in another PEC system where BiVO_4_ was used. According to the results obtained, in the absence of PMS, 55.2 % of ofloxacin was degraded under 120 min. Howbeit, when 2 mM PMS was introduced, the ofloxacin was almost completely degraded. This increased performance was attributed increased production of sulphate radicals [37]. Also, this was further corroborated by Zhang et al. where increased PMS concentration gave rise to increased BPA degradation efficiency. In the absence of PMS, only 5.88 % of BPA was degraded under the PEC system within 60 min. In contrast, on increasing the PMS concentration from 0.5 to 2 mM, 99.16 % of the BPA was degraded within 60 min, hence indicating the significant role of sulphate radicals in the PEC abatement of BPA [38]. We can therefore clearly state that the more sulphate radicals present in a PEC system, the greater the extent of degradation of organic pollutants present in the system. The degree of mineralisation of tetracycline as estimated from the results obtained from the total organic carbon (TOC) was 75 %.

The roles of visible light and WO_3_ NRs towards the generation of sulphate radicals were investigated by monitoring the extent of degradation of tetracycline using FTO only and FTO-WO_3_ NRs in the presence of visible light. When the FTO only was exposed to light, the extent of tetracycline degradation was found to be 43 % (Fig. 4a). This shows that visible light can activate persulphate salt to generate sulphate radicals. However, when compared to FTO-WO_3_ NRs, the extent of tetracycline rose to 88 %. This notable difference was as a result of the photogenerated electrons present at the valence band of the WO_3_ NRs semiconductor. These electrons activated persulphate salt thereby producing more sulphate radicals for the abatement of tetracycline. Hence, the improved photoelectrocatalytic degradation of tetracycline can be attributed to the photoelectrocatalytic synergistic reaction taking place between WO_3_ NRs and sulphate radicals in the presence of visible light. A similar trend has been previously reported in one of our works [8].

The apparent rate constant for the breakdown of tetracycline in the absence and presence of PS was estimated from the plot of the linearised pseudo first order kinetic model as presented in Fig. 4b. The result obtained followed was consistent with what was obtained in Fig. 4a. The rate constant for the PEC system was 0.00138 min^−1^, while in the presence of 1 mM PS, the apparent rate constant was 0.00550 min^−1^, which was nearly 4 times faster than the PEC system. Interestingly, the rate constant increased to 0.02341 min^−1^ in the presence of 3 mM PS, which was nearly 14 times faster than the PEC system.

The increase in the extent of breaking down tetracycline in the PEC/PS system is because of the photogenerated charge carriers. While the photogenerated electrons activated the persulphate salt in the presence of light to generate more sulphate radicals for the degradation of tetracycline, the photogenerated holes present in the valence band also reacted with the tetracycline, thereby breaking them down in the system [39].

#### 3.3.1 Influencing factors on the photoelectrocatalytic degradation of tetracycline

The potential applied significantly contributes towards any degradation process as it impedes the extent of recombination of the photogenerated electron and holes, thereby providing mobility for the holes to react with the pollutant. Also, the photogenerated electron will activate the PS salt to generate sulphate radicals for the enhanced degradation of the pollutant. The PEC/PS degradation of tetracycline experiment was conducted by varying the potential within 0.5–1.5 V as shown in Supp. Fig. S2a. It was discovered that the extent of degradation of tetracycline rose from 73 % at 0.5 V to 88 % at 1.5 V. This is because the higher the potential, the better the charge carrier separation thereby resulting in improved degradation efficiency. This result was further substantiated by the kinetics study as the rate constant at 1.5 V was almost twice that of 0.5 V (Supp. Fig. S2b).

It is of great importance to state here that the results produced by the PEC/PS system employed in our work agree with the results obtained by other reports according to literature. Orimolade et al. [7] carried out a study on PMS-assisted PEC degradation of tetracycline among other pharmaceuticals at an FTO-Bi_2_WO_6_ photoanode. Their results showed 77 % removal of tetracycline within 90 min compared to 88 % tetracycline removal recorded in our work.

In addition, the influence of the pH of the solution in the photoelectrocatalytic abatement of tetracycline was investigated at pH 3.1, 6.7 and 9.1 as shown in Supp. Fig. S2c. From our result, 74 % of the tetracycline was degraded in the acidic medium (pH = 3.1). As the pH of the solution was increased to the neutral medium (pH = 6.7), 88 % of the tetracycline was degraded. However, in the basic medium (pH = 9.1), the extent of tetracycline degradation dropped to 70 %. This shows the FTO-WO_3_ NRs electrode in the presence of sulphate radicals will efficiently degrade effluents containing antibiotics in the acidic and neutral medium. This result agrees with previous studies on the effect of pH on the abatement of tetracycline [40].

#### 3.3.2 Degradation studies, intermediate products, degradation pathways, and ecotoxicity

The extent of degradation of tetracycline was also monitored using UPLC-MS as aliquots were drawn at time intervals between 0 and 90 min. The percentage degradation obtained was found to be 99 %. This result obtained agreed with the same trend obtained when the extent of degradation was measured with a UV–vis spectrophotometer. However, the higher value obtained using UPLC-MS is due to the fact that it is a more sensitive technique than the UV–vis spectrophotometer.

**Fig. 5:**
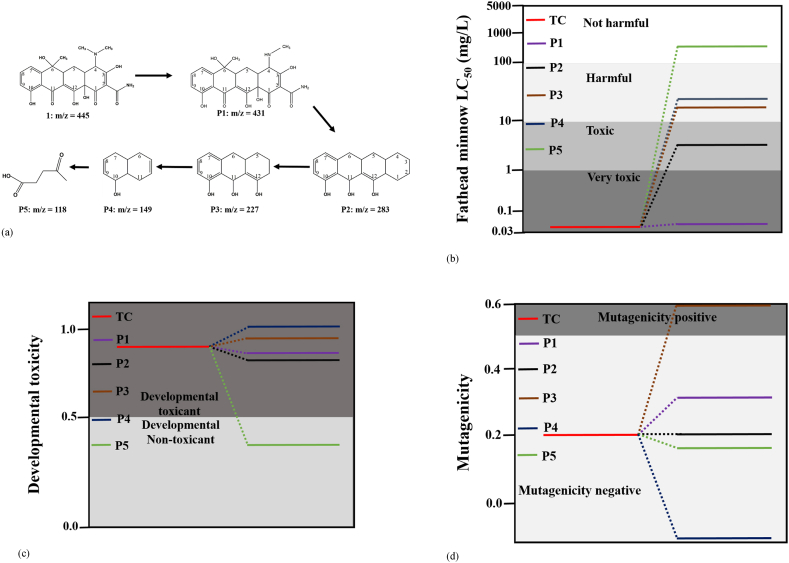
(a) Proposed degradation pathway and intermediates products during the PEC degradation of tetracycline on FTO-WO_3_ NRs photoanode, (b) Fathead minnow LC_50_, (c) developmental toxicity, (d) mutagenicity.

To confirm the intermediate products formed and propose possible degradation pathways of tetracycline on the FTO-WO_3_ NRs photoanode, UPLC-MS analysis of the tetracycline standard as well as the aliquots drawn at intervals was carried out. The chromatograms obtained at different time intervals are shown in Supp. Figs. S3(a–d). A peak 3.27 min (*m*/*z* = 445) was detected for tetracycline standard. Also, the intermediate products generated during the degradation of tetracycline within 90 min were identified from the different ion peaks detected and these are shown in Fig. 5a.

The probable degradation pathway is represented in Fig. 5a. In the degradation of tetracycline, the demethylation process played a vital role. This is because of the lesser bond energy of the N–C bond [41]. There is a possibility of a demethylation reaction taking place as the dimethylamino moiety on the carbon position 4 gets attacked by hydroxyl radicals to form an intermediate product, P1 (*m*/*z* = 431). This was followed by a series of reactions such as deamination and dehydroxylation to produce an intermediate product, P2 (*m*/*z* = 283). Furthermore, intermediate products P4 and P5 with peaks at *m*/*z* = 149 and 118 respectively were possibly formed by the cleavage of carbon-carbon bond and ring-opening reactions. A similar trend has been reported [29,41].

To investigate the toxicity level of tetracycline and its intermediate products in the environment, the acute toxicity, developmental toxicity and mutagenicity of tetracycline were estimated using the Quantitative Structure Activity Relationship (QSAR) model by using the Toxicity Estimation Software Tool (T.E.S.T). As represented in Fig. 5b and Table 1, the LC_50_ of fathead minnow, shows the concentration of tetracycline that is present in water which can cause death to half of the exposed fathead minnows (*Pimephales promelas*) within 4 days/96 h. The value obtained for tetracycline in this work is 0.0697 mg/L. This value can be said to be “very toxic” because according to the literature, 0–1 mg/L LC_50_ value can be said to be very toxic, 1–10 mg/L can be said to be toxic, 10–100 mg/L can be said to be harmful, and >100 mg/L is not harmful [42,43]. Also, the demethylated intermediate product P1 was still “very toxic” from the estimated LC_50_ value of 0.0698 mg/L. Further degradation of the intermediates led to a reduction in the acute toxicity as product P2 was found to be toxic and products P3 and P4 were “harmful”. Interestingly, the LC_50_ value for product P5 was 435.56 mg/L indicating that it is “not harmful”. Furthermore, according to the developmental toxicity test, tetracycline and intermediate products P1, P2, P3, and P4 were all considered to be “toxicant” as they all showed values greater than 0.5 indicating that they will interfere with normal development both before and after birth in humans and/or animals. It is important to note that the product P5 can be considered to be “non-toxicant” with a value less than 0.5 (Fig. 5c) showing that there will be no interference in the development of humans and/or animals before and after birth. For mutagenicity studies, a compound is said to be positive when the value obtained is greater than 0.5, and negative when the value obtained is less than 0.5. As seen in Fig. 5d as well as Table 1, the tetracycline and products P1, P2, P4, and P5 were all mutagenicity negative except P3 which can be said to be positive suggesting that it can significantly cause revertant colony growth. Overall, it can be seen that some intermediate products are still toxic. Nevertheless, we have successfully reduced their level of toxicity compared to the parent compound and we were able to produce a final product that is neither harmful nor developmentally toxicant.

**Table 1:** Toxicity prediction values and results of TC and its intermediates calculated by T.E.S.T.

Name	Fathead minnow LC_50_ (96 h) (mg/L)	Developmental toxicity		Mutagenicity	
		Predicted value	Predicted result	Predicted value	Predicted result
TC	0.0697	0.85	Developmental toxicant	0.20	Negative
P1	0.0698	0.81	Developmental toxicant	0.29	Negative
P2	5.16	0.79	Developmental toxicant	0.20	Negative
P3	18.49	0.93	Developmental toxicant	0.60	Positive
P4	24.95	1.02	Developmental toxicant	−0.03	Negative
P5	435.56	0.40	Developmental non-toxicant	0.18	Negative

### 3.4 Scavenger studies and reusability test

**Fig. 6:**
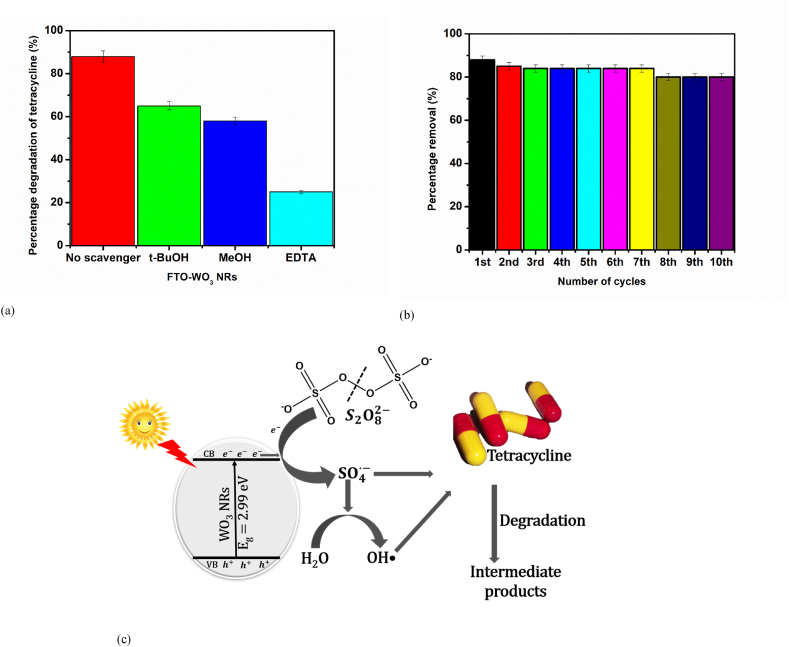
(a) Trapping experiment for the PEC degradation of tetracycline, (b) Stability test for the degradation of tetracycline at FTO-WO_3_ NRs photoanode, (c) schematic diagram of the activation of PS for enhanced degradation of tetracycline.

To gain a better understanding of the part played by the oxidising species in the photoelectrocatalytic degradation process, we carried out scavenger studies to trap oxidants such as hydroxyl radicals, sulphate radicals, and photogenerated holes. The tertiary butanol (t-BuOH) was used to mask the hydroxyl radicals, methanol (MeOH) was used to trap both sulphate and hydroxyl radicals and sodium salt of ethylenediaminetetraacetic acid (EDTA) was used to scavenge the photogenerated holes. As illustrated in Fig. 6a, the breakdown of tetracycline decreased to 58 % in the presence of MeOH confirming the significant roles of both sulphate and hydroxyl radicals in the breakdown of tetracycline. Also, the abatement of tetracycline decreased to 65 % in the presence of t-BuOH and 25 % in the presence of EDTA. The marked reduction in the extent of degradation when EDTA was added showed that the holes was majorly responsible for the photoelectrocatalytic degradation of tetracycline.

Furthermore, it is expected of an excellent photoanode to be highly stable and reusable. Therefore, we examined the extent to which the FTO-WO_3_ NRs photoanode can be said to be stable and reusable by using it for the PS-assisted PEC degradation of tetracycline as shown in Fig. 6b. The electrode was fairly stable after 10 cycles as the percentage degradation decreased from 88 % to 80 %. We must state that this result confirms that the photoanode showed great stability and reusability as each cycle of the experiment takes 90 min.

Due to the results obtained from the scavenger studies, the mechanism of peroxydisulphate activated FTO-WO_3_ nanorods for improved degradation of tetracycline is represented in eqns. (5)–(10) as well as Fig. 6c.

(5) $${\text{WO}}_{3}+hv\;\to \;{\text{WO}}_{3}\;\left(e_{CB}^{-}+h_{VB}^{+}\right)$$

(6) $${\text{WO}}_{3}\;\left(e_{CB}^{-}\right)+S_{2}O_{8}^{2-}\;\to \;SO_{4}^{2-}+SO_{4}^{∙-}$$

(7) $$SO_{4}^{∙-}+\text{tetracycline}\;\to \;\text{Intermediate}\;\text{products}$$

(8) $$h_{VB}^{+}+\text{tetracycline}\;\to \;\text{Intermediate}\;\text{products}$$

(9) $$h_{VB}^{+}/SO_{4}^{∙-}+H_{2}O\;\to \;SO_{4}^{2-}+\text{OH}•$$

(10) OH•+tetracycline→Intermediateproducts

## 4 Conclusion

Tungsten trioxide nanorods were successfully prepared via the hydrothermal method and conducted on a FTO glass substrate to form a photoanode. UV–vis diffuse reflectance spectroscopy confirmed the material to be visible light active. The addition of sulphate radicals into the PEC setup significantly enhanced the degradation of tetracycline as 88 % of the tetracycline was degraded as obtained from UV–vis spectrophotometer data. In addition, results obtained from the ultra-performance liquid chromatography mass spectrometer showed that 99 % of the tetracycline was degraded within 90 min at a potential of 1.5 V. The electrode was stable and reusable. The intermediate products identified during the PEC degradation process provided insight into the elucidation of their structures and was used in proposing a possible tetracycline degradation pathway. The obtained toxicity result was based on the removal of the parent compound. Placing emphasis on toxicity that result from the degradation of the parent compound alone can be misleading, hence we recommend that attention should be given to investigating the level of toxicity of the intermediate products formed during the degradation process.

## Author contribution statement

Babatunde A. Koiki: Conceived and designed the experiments; Performed the experiments; Analyzed and interpreted the data; Wrote paper.

Omotayo Ademola Arotiba: Conceived and designed the experiments; Analyzed and interpreted the data; Contributed reagents, materials, analysis tools or data.

## Data availability statement

Data will be made available on request.

## Declaration of competing interest

The authors declare that they have no known competing financial interests or personal relationships that could have appeared to influence the work reported in this paper.
