# Supplementary material for: Peroxydisulphate activated FTO-WO3 nanorods based photoelectrocatalytic degradation of tetracycline: Intermediate products, degradation pathway and ecotoxicity studies
Source: Heliyon. 2023 Oct 12;9(10):e20882. doi: 10.1016/j.heliyon.2023.e20882 (PMC10590805; doi:10.1016/j.heliyon.2023.e20882)
Supplement: Multimedia component 1 [file mmc1.docx]

**Peroxydisulphate activated FTO-WO_3_ nanorods based photoelectrocatalytic degradation of tetracycline: intermediate products, degradation pathway and ecotoxicity studies**

Babatunde A. Koiki^a^, Omotayo A. Arotiba^a,b*^

*^a^Department of Chemical Sciences, University of Johannesburg, South Africa*

*^b^Centre for Nanomaterials Science Research, University of Johannesburg, South Africa*

**Corresponding author: Email address:* [*oarotiba@uj.ac.za*](mailto:oarotiba@uj.ac.za)

**Supplementary Information**





(a)

(b)



**Fig. S1.** (a) UV- vis diffuse reflectance spectra of WO_3_ NRs (b) Tauc plot showing the band-gap energy of WO_3_ NRs.





(a)





(b)





(c)

**Fig. S2.** (a) Effect of bias potential on PEC degradation of tetracycline, (b) apparent reaction rate constant, (c) Effect of solution pH on PEC degradation of tetracycline. Experimental conditions: [Tetracycline] = 5 ppm, [PS] = 3 mM


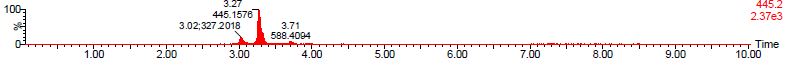


(a)


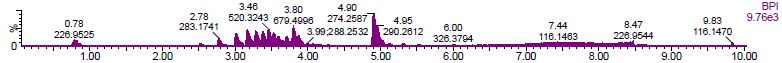


(b)


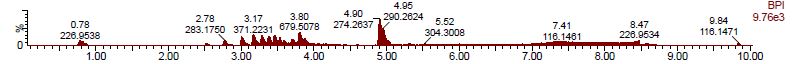


(c)


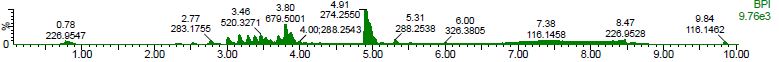


(d)

**Fig. S3.** UPLC-MS chromatograms of (a) tetracycline standard solution, (b) aliquot taken after 30 min, (c) aliquot taken after 60 min, (d) aliquot taken after 90 min.
